# Statistical Dissection of the Genetic Determinants of Phenotypic Heterogeneity in Genes with Multiple Associated Rare Diseases

**DOI:** 10.3390/genes14112100

**Published:** 2023-11-18

**Authors:** Tatyana E. Lazareva, Yury A. Barbitoff, Yulia A. Nasykhova, Nadezhda S. Pavlova, Polina M. Bogaychuk, Andrey S. Glotov

**Affiliations:** 1 Department of Genomic Medicine, D.O. Ott Research Institute of Obstetrics, Gynaecology, and Reproductology, 199034 St. Petersburg, Russia; lazata1997@gmail.com (T.E.L.); yulnasa@gmail.com (Y.A.N.); 2 Bioinformatics Institute, Kantemirovskaya St. 2A, 197342 St. Petersburg, Russia; pav.nad.ser@gmail.com (N.S.P.); pm.bogaichuk@gmail.com (P.M.B.)

**Keywords:** rare disease, genetic variants, phenotypic heterogeneity, variant localization, variant interpretation

## Abstract

Phenotypicheterogeneity is a phenomenon in which distinct phenotypes can develop in individuals bearing pathogenic variants in the same gene. Genetic factors, gene interactions, and environmental factors are usually considered the key mechanisms of this phenomenon. Phenotypic heterogeneity may impact the prognosis of the disease severity and symptoms. In our work, we used publicly available data on the association between genetic variants and Mendelian disease to investigate the genetic factors (such as the intragenic localization and type of a variant) driving the heterogeneity of gene–disease relationships. First, we showed that genes linked to multiple rare diseases (GMDs) are more constrained and tend to encode more transcripts with high levels of expression across tissues. Next, we assessed the role of variant localization and variant types in specifying the exact phenotype for GMD variants. We discovered that none of these factors is sufficient to explain the phenomenon of such heterogeneous gene–disease relationships. In total, we identified only 38 genes with a weak trend towards significant differences in variant localization and 30 genes with nominal significant differences in variant type for the two associated disorders. Remarkably, four of these genes showed significant differences in both tests. At the same time, our analysis suggests that variant localization and type are more important for genes linked to autosomal dominant disease. Taken together, our results emphasize the gene-level factors dissecting distinct Mendelian diseases linked to one common gene based on open-access genetic data and highlight the importance of exploring other factors that contributed to phenotypic heterogeneity.

## 1. Introduction

Next-generation sequencing (NGS) is increasingly being utilized in biomedical research as a powerful tool for identifying causal genetic variation behind hereditary diseases. NGS results are also important for the selection of targeted therapies, prediction of treatment responses for cancer, and evaluation of individual drug sensitivity. Numerous genetic variants are discovered in a single NGS-based experiment; however, identifying the causal variant(s) for a certain phenotype is a serious challenge in medical genetics. Thus, ∼40% of variants are still classified as variants of uncertain significance according to internationally accepted recommendations developed by the American College of Medical Genetics (ACMG) (reviewed in [1]). The clinical classification of genetic variants includes rigorous criteria for assessing their pathogenicity, and these criteria are based on allele frequency data, familial segregation studies, results of computational prediction of pathogenicity, and functional evidence, such as variant location in functionally crucial domains [2]. To clarify the recommendations and improve exception management, refined sets of ACMG-derived guidelines have been proposed by Sherloc [3] and the Clinical Genome Resource Sequence Variant Interpretation Working Group [4].

Phenotypic heterogeneity is one of the complicating factors of variant interpretation. The term “phenotypic heterogeneity” refers to the variation in disease severity (or even the development of distinct diseases) in individuals carrying pathogenic variants in the same gene (or even the same genotype). The phenotypic heterogeneity has been observed in phenotypically distinct groups of disorders. Notable examples include beta-thalassemias [5] or different types of cardiomyopathies caused by mutations in *ANKRD1* [6]. In cystic fibrosis, for example, individuals with the same mutations in the causal *CFTR* gene can exhibit varying degrees of lung disease and pancreatic insufficiency [7]. In another notable case, divergent clinical manifestation has been observed even within family members with Brugada syndrome [8].

A particular subtype of phenotypic heterogeneity is the phenomenon of multiple diseases associated with the same gene, which we denote as heterogeneous gene–disease relationships. In some cases, a single gene can be linked to more than 5 diseases. An example of such a gene is *LMNA* which encodes nuclear lamin A/C, an important structural component involved in a wide range of molecular processes from nuclear and chromatin organization to DNA repair [9]. There are as many as 11 distinct conditions linked to *LMNA*, including muscular dystrophies (OMIM:181350, OMIM:616516, OMIM:613205), inherited cardiac conditions (OMIM:115200) involving forms associated with hypergonadotropic hypogonadism (OMIM:212112) or brachydactyly (OMIM:610140), genodermatosis (OMIM:619793), neuropathy (OMIM:605588), lipodystrophy (OMIM:151660, OMIM:248370) and premature aging disorders (OMIM:176670).

The medical community still lacks a consensus regarding lumping diverse phenotypic observations associated with the same genotype into one disease with sub-types or splitting into independent nosological entities to better reflect the disease’s nature. Several criteria were proposed by Thaxton et al. to decide individual cases [10]. The suggestions include the comparison of sets of phenotype features between family members and reviewing previous findings, as well as estimating the inheritance modes and molecular mechanisms of observed conditions.

Penetrance, expressivity, and genetic pleiotropy are among the underlying contributing factors to phenotypic heterogeneity, arising from various causes, including genetic, epigenetic, and environmental influences (reviewed in [11,12]). However, it is not clear what features of the genes are correlated with the observed heterogeneity. At the level of individual genetic variants, variant localization and type might be important for phenotype determination in genes linked to multiple diseases. For example, variants in *CDC42* located in different parts of this gene can disturb the intracellular signaling function of the gene product in different ways, thus causing various developmental phenotypes [13]. The connection between mutation type and phenotype severity can be well illustrated by the *GLUT1* deficiency syndrome, in which milder symptoms develop in patients with missense mutations in contrast to more severe phenotype resulting from causal predicted loss-of-function (pLoF) variants [14].

The dissection of variant features that play a role in gene–disease relationships is important for a better understanding of the disease’s nature and more precise clinical diagnosing. However, no systematic analysis of the relative importance of the above-mentioned gene-level factors has yet been performed on publicly available datasets. Hence, in this study, we explored common features of genes linked to multiple rare diseases and properties of genetic variants that might explain the phenomenon of phenotypic heterogeneity in such genes.

## 2. Materials and Methods

### 2.1. Data Collection

Information about the association between human genes and rare diseases, inheritance patterns, and phenotypic terms associated with each disease was retrieved from the Human Phenotype Ontology (HPO) [15]. For further analysis, polygenic, somatic diseases, and provisional gene–disease associations were filtered out.

For the analysis of constraint, we obtained Loss-of-function Observed/Expected Upper Fraction (LOEUF) scores from the Genome Aggregation Database (gnomAD) gene-level summary statistics [16]. These scores were pre-ranked and binned into deciles ranging from 0 (most pLoF-depleted/constrained) to 9 (not pLoF-depleted/constrained). Additionally, the probability of Loss-of-function Intolerance (pLI) was used to separate genes into LoF-tolerant (pLI ≤0.1) or intolerant (pLI ≥0.9). Genes with 0.1 < pLI < 0.9 were considered to be ones with uncertain LoF-tolerance status.

For isoform expression analysis, we acquired the Genotype-Tissue Expression (GTEx) V8 transcript expression dataset from the GTEx web portal [17]. The median gene expression level was employed to assess the number of expressed isoforms (expression value of TPM > 5 in at least one tissue was used as a cutoff to designate the expressed transcripts).

Information about the genetic variants linked to rare diseases was obtained from the NCBI ClinVar database. We extracted all variants marked as pathogenic (P) and likely pathogenic (LP) in ClinVar as of 3 September 2023 [18]. The relationship between each variant and disease was ascertained using the OMIM identifier provided for each variant by ClinVar.

### 2.2. Gene-Set Enrichment Analysis

To explore shared biological processes and phenotypic traits for genes with multiple diseases, we conducted an enrichment analysis using the clusterProfiler R package [19]. We utilized the Molecular Signatures Database (MSigDB) collections for *H. sapiens* (canonical pathways (C2.CP) and Human Phenotype Ontology (C5.HPO)). Term–gene associations were retrieved by the msigdbr v. 7.5.1 R package. All genes included in our dataset were used as a universe during enrichment testing.

### 2.3. Phenotypic Similarity Analysis

For each pair of diseases associated with a single gene, we estimated the similarity of phenotypic features denoted in HPO terms. The terms were lifted to upper-level HPO terms (i.e., direct descendants of phenotypic abnormality) by a custom script, and the Jaccard index was estimated using sets of upper-level terms for each pair of diseases. The ontobio package in Python 3.9 was used for this purpose (https://github.com/biolink/ontobio (accessed on 28 September 2023)). Similarity scores for random pairs of disorders linked to different genes were used as a baseline for comparison.

### 2.4. Statistical Analysis of Within-Gene Distribution and Type of Variants

We conducted a statistical analysis of the within-gene distribution of variants to determine the impact of variant localization in the coding sequence on the development of different diseases associated with a single gene. The analysis was restricted to genes with two linked diseases. Two different versions of this analysis were conducted: (i) splitting a gene’s CDS into a fixed number of intervals of equal size and (ii) using the annotated protein domain boundaries.

In the first case, we obtained a list of canonical transcripts of selected genes from Ensembl BioMart [20] and collected the CDSs regions annotated for the canonical transcript in GENCODE v. 44 genome annotation. We then divided each coding transcript sequence into n intervals of equal length and used the BedTool package for Python to intersect each interval with locations of ClinVar P/LP variants linked to each of the diseases separately. This resulted in a table with the number of variants in each bin associated with each of the two diseases. We then filtered out genes that had less than five variants associated with each of the traits.

For the second version of the analysis, the UniProt domain mapping to the reference genome was obtained from the UCSC table browser [21]. Genes with less than 2 and more than 11 domains were filtered out, and P/LP variants were counted within each domain. Fisher’s exact test was used to assess the significance of the differences in the location of variants for diseases.

Additionally, we performed a statistical comparison of the proportion of variants of different types (i.e., missense or pLoF) linked to different diseases in the same gene. To do so, we calculated the total number of missense and pLoF (i.e., stop gained, splice site, or frameshift) variants for each disease and compared them using Fisher’s exact test.

### 2.5. Data and Code Availability

All data and code pertinent to the analysis presented in this work are available at https://github.com/tanya-lazareva/phenohet.git (accessed on 8 October 2023).

## 3. Results

### 3.1. Common Properties of Genes Linked to Multiple Rare Diseases

**Figure 1:**
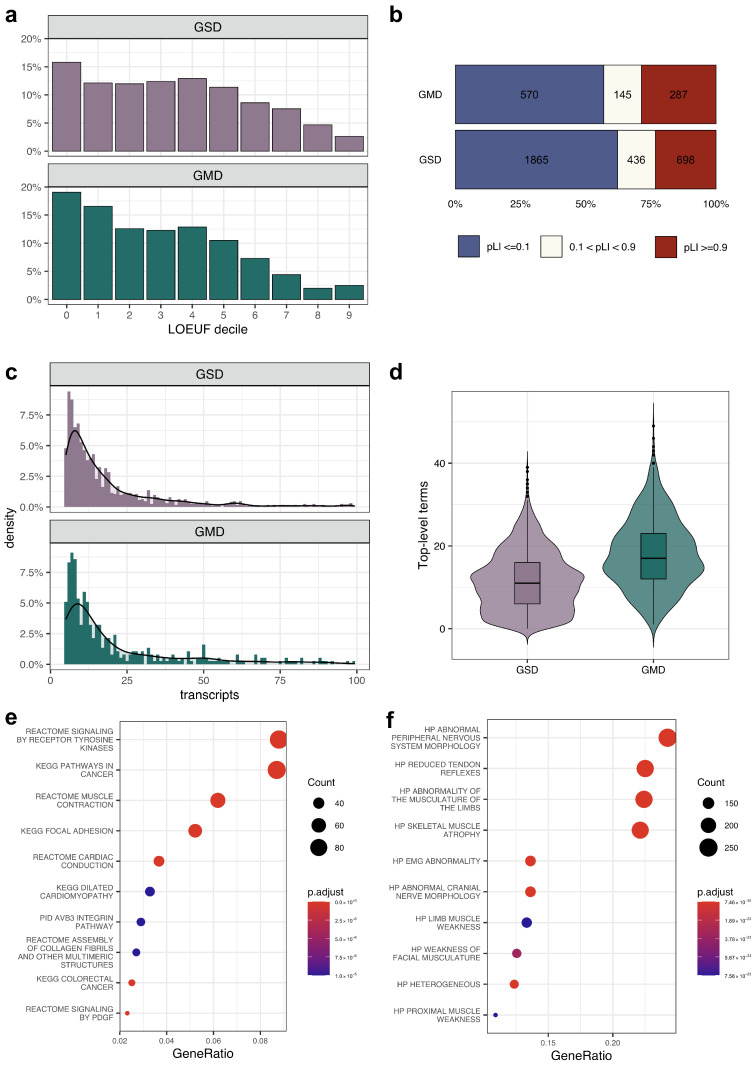
General description of genes linked to multiple genetic disorders. (**a**) A histogram showing the number of genes in each LOEUF decile among genes with 1 (GSD) or with 2 and more associated (GMD) rare diseases. (**b**) Proportion of genes that are tolerant (pLI ≤ 0.1) or intolerant (pLI ≥ 0.9) to loss-of-function mutations for genes linked to strictly 1 disease or 2 and more diseases. (**c**) Histogram showing the distribution of the number of expressed transcripts (>5 TPM in at least one tissue according to the Genotype-Tissue Expression (GTEx) data) for GSDs and GMDs. (**d**) Plots representing the number of upper-level Human Phenotype Ontology (HPO) terms annotated for the two groups of genes. (**e**,**f**) Dot plots showing the results of enrichment analysis of genes linked to 2 or more rare diseases. Enrichment analysis against genes from MSigDB-derived canonical pathways (**e**) or HPO terms (**f**) are shown.

To understand the genetic basis of phenotypic heterogeneity in rare diseases, we first attempted to identify the common features of genes linked to multiple distinct disorders. To conduct such an analysis, we started by collecting data on gene–disease associations from the HPO database. In total, we identified 4211 genes that are related to 5671 Mendelian diseases from OMIM. Interestingly, 18.3% (1035) of those exhibit associations with two or more conditions. We next went on to compare these genes (denoted as Genes with Multiple associated Diseases, GMD) to genes linked to exactly 1 rare disease (denoted as Genes with a Single associated Disease, GSD). We first compared the degree of constraint of these genes using the pLI and LOEUF metrics provided by gnomAD. The comparison showed that a greater proportion of GMDs are sensitive (or intolerant) to pLoF variants compared to GSDs (Figure 1b) (*p*-value in chi-squared test = $5.5\times 10^{-5}$). Similarly, the distribution of LOEUF for genes with phenotypic heterogeneity, as illustrated on Figure 1a, also shows a greater degree of constraint for these genes. It suggests that GMDs are subjected to greater constraints and are potentially involved in a broad range of biological processes. Furthermore, the isoform expression analysis revealed that the number of expressed transcript isoforms is consistently higher for GMDs (Figure 1c) (Wilcoxon test, *p*-value = $9.5\times 10^{-11}$). At the next stage of the analysis, we compared the total number of upper-level human phenotype terms described for GMDs and GSDs. Figure 1d shows that GMDs are associated with more upper-level HPO terms compared to GSDs. This observation also confirms that variants in GMDs have a greater impact on the human phenotype, in line with the greater functional importance of these genes.

We then tried to identify the biological processes and disease groups associated with GMDs. To do so, we performed a gene-set enrichment analysis of the complete list of GMDs using gene annotations from MsigDB (see Section 2). The enrichment analysis revealed that GMDs are overrepresented in gene sets associated with oncogenesis, muscle contraction (including heart muscle contraction), tyrosine kinases receptor-mediated, and platelet-derived growth factor signaling pathways (Figure 1e). In concordance with these observations, a set of GMDs was enriched with genes associated with neuromuscular abnormalities (Figure 1f).

**Figure 2:**
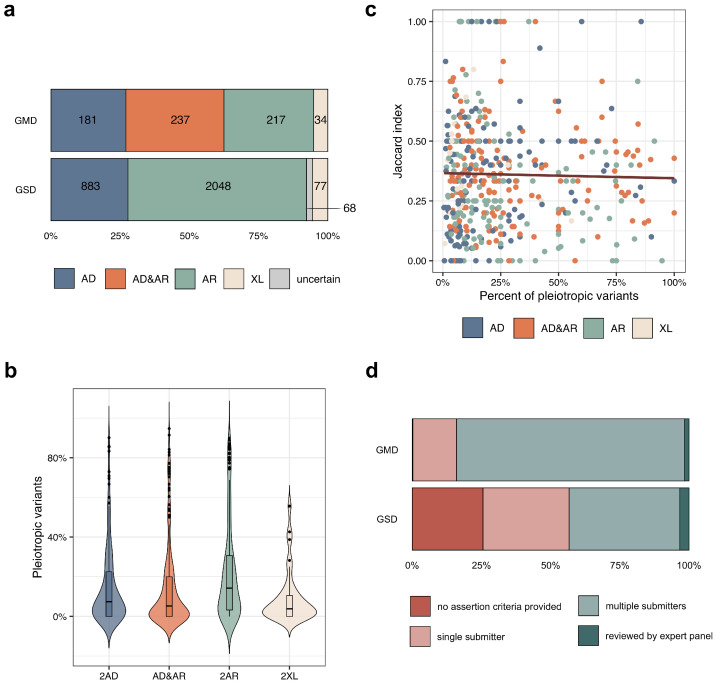
The proportion of shared pathogenic variants does not correlate with inheritance patterns or phenotypic similarity of the diseases linked to GMDs. (**a**) A bar plot showing the number of genes linked to two autosomal recessive (AR), one autosomal dominant and autosomal recessive (AD&AR), two autosomal dominant (AD), X-linked (XL) diseases and diseases with uncertain inheritance mode in GMDs and GSDs. (**b**) Plots representing the proportion of variants linked to both diseases for genes in each inheritance pattern group. (**c**) Correlation between the phenotypic similarity between the two diseases linked to the same gene in each inheritance pattern group and abundance of P/LP variants described for two disorders. (**d**) Review status of P/LP ClinVar variants associated with one or both diseases linked to genes with exactly 2 described rare disorders.

Next, we explored the inheritance pattern of diseases associated with GMDs, focusing on genes with exactly 2 associated diseases. We split genes into 4 groups based on the inheritance pattern of the associated diseases (i.e., 2AD—both diseases have autosomal dominant (AD) inheritance mode, 2AR—both are autosomal recessive (AR), AD&AR—the two diseases have different inheritance patterns (AD and AR), and 2XL—genes with X-linked inheritance (all genes located on the X chromosome were grouped due to their relatively low number)). As can be seen from Figure 2a, the number of genes with mixed inheritance patterns (AD&AR) is comparable to the number of ones with 2AD and 2AR inheritance modes of associated diseases. This finding suggests that differences in inheritance patterns could account only for a fraction of phenotypic heterogeneity in GMDs, and other factors are also involved in phenotype determination. At the same time, the proportion of strictly AR genes is significantly decreased among GMDs compared to GSDs, corroborating the results of constraint analysis (Figure 1b).

During our analysis, we noticed that pathogenic (P) and likely pathogenic (LP) variants linked to both diseases in a GMD have been described in ClinVar for 458 genes. We hypothesized that these variants, termed “pleiotropic”, could be prevalent in genes associated with two diseases that possess distinct inheritance patterns, namely AD and AR. In other words, if the zygosity of the pathogenic allele is important for phenotype specification, more pathogenic alleles should be shared between the two diseases in the AD&AR genes. However, the proportion of shared pathogenic alleles was the greatest for the 2AR genes (Figure 2b). This suggests that the abundance of “pleiotropic” variants does not depend on the inheritance pattern of the associated disorders. We next hypothesized that the share of pleiotropic variants should be higher for GMDs with greater phenotypic similarity of the corresponding diseases. However, as can be seen in Figure 2c, no correlation was observed between phenotypic similarity and the percent of shared P/LP variants between pairs of monogenic disorders linked to one GMD. Therefore, we can assume that the abundance of “pleiotropic” variants can be explained by inaccuracies in variant annotation in open-access datasets, despite the fact that most P/LP variants in GMDs are supported by assertion criteria of from multiple submitters (Figure 2d).

### 3.2. The Effects of Variant Localization and Type on the Phenotypic Outcome

Having characterized the properties of GMDs and the associated disorders, we next went on to assess the contribution of different genetic factors to phenotypic heterogeneity in such genes. We decided to limit our analysis to two clearly defined properties of a genetic variant: its intragenic localization and functional type.

**Figure 3:**
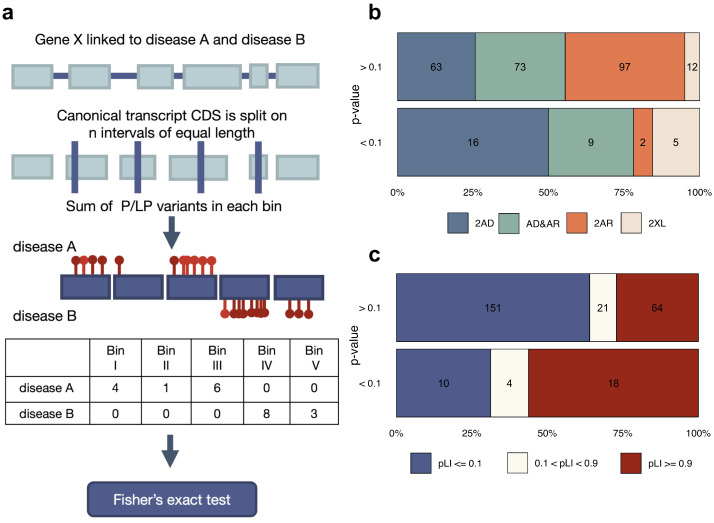
The influence of the variant localization on the observed phenotype. (**a**) A schematic illustration of the workflow of analysis of variant distribution within the coding sequence of the genes with 2 associated diseases. (**b**,**c**) The proportion of genes in different inheritance pattern categories (2AD, AD&AR, 2AR, 2XL) (**b**) or different LoF-tolerance groups (**c**) for genes with or without weak nominally significant (*p* < 0.1) differences in the distribution of variants for the two associated diseases.

Different parts of the same gene may encode distinct functional domains of the protein product. Consequently, the impact of variants located in various domains may differently influence the development of particular phenotypes [22]. To examine the effect of variant localization within coding sequence on disease development, we analyzed GMDs linked to exactly 2 conditions that have 5 or more variants annotated as causal for each disease in ClinVar. As a first step in our analysis, we evaluated the effect of variant localization by splitting the CDS of a gene into 5 equally sized bins and calculating the number of variants in each bin (Figure 3a, see Section 2 for a more detailed description of the procedure).

Inspection of the results of the test using quantile-quantile plot (Supplementary Figure S2a) showed only a slight deviation from the expectation, suggesting that the effects of variant localization are either low or masked by other confounding factors. At the same time, we managed to identify several genes that reached FDR-adjusted statistical significance in the test, namely *CDKN1C*, *CREBBP*, *OCRL*, and *SPTB*. Moreover, when pleiotropic (i.e., linked to both diseases) variants were removed, much greater inflation of the test statistic was observed (Supplementary Figure S2b), and 13 genes reached FDR-adjusted significance. This observation suggests that the effects of variant localization are pronounced, but masked by noise in variant–disease annotations (Table 1, Supplementary Table S2).

We next went on to investigate which properties of the genes correlate with the effect of variant localization on phenotype. To this end, we split all GMDs into two groups based on the nominal *p*-value of the variant localization test. Such analysis showed that 2AD genes, as well as more constrained genes according to pLI (Figure 3b,c), appear to be more abundant in the group GMDs with a significant effect of variant localization. Given this relationship between the AD inheritance pattern and the impact of variant localization on the phenotype, we next compared the phenotypic similarity between diseases linked to GMDs in the 2AD inheritance pattern category. Remarkably, diseases linked to genes with significant effects of variant position exhibited lower similarity (Supplementary Figure S3). This result suggests that different AD diseases caused by variants in different parts of the same gene tend to have different phenotypic manifestations.

Given the aforementioned result, we next questioned whether a more significant effect of variant localization on phenotypic heterogeneity can be observed when splitting the transcript length into two parts instead of five. Our analysis revealed that splitting the coding region into two parts did not result in significant differences compared to the previously described 5-bin test. However, four genes (*ATM*, *DHH*, *EYA4*, *NALCN*) showed significant differences in the localization of causal variants for the two associated diseases only when the coding sequence was split into two parts. At the same time, the significance of variant localization differences was lost for 14 genes (*CENPJ*, *DEAF1*, *DMD*, *HTRA1*, *KCNQ2*, *KLHL7*, *MADD*, *NOTCH1*, *NOTCH2*, *OCRL*, *ROR2*, *SETBP1*, *SPTBN2*, *STUB1*) when splitting the CDS into two parts instead of five (Supplementary Figure S2c). This suggests that more complex gene structures rather than N- and C-terminal regions might be involved in the pathogenesis of different diseases linked to GMDs.

**Figure 4:**
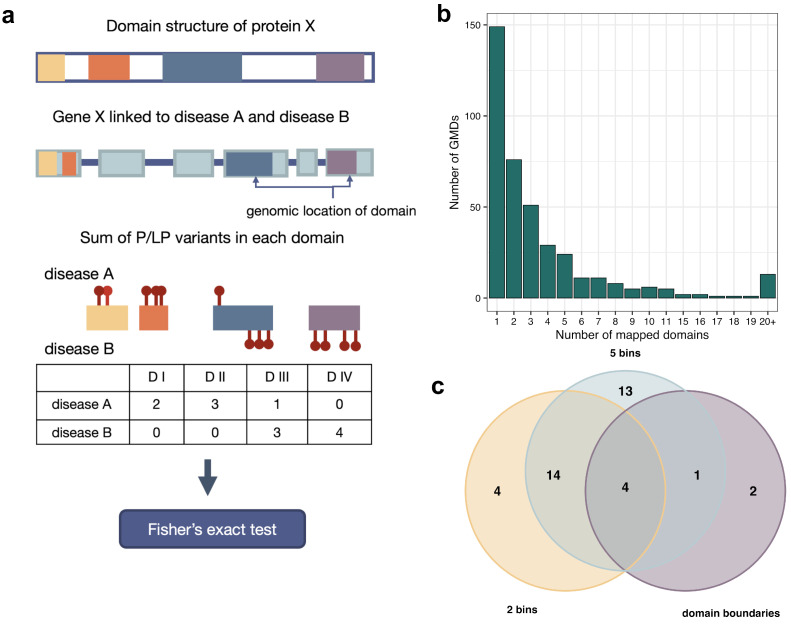
The influence of the variant distribution within protein domains on the observed phenotype. (**a**) A schematic illustration of the workflow of analysis of variant distribution within protein domains in GMDs with exactly 2 associated diseases. (**b**) Histogram showing the number of domains annotated to coordinates of GMDs with exactly 2 associated conditions. (**c**) Venn diagram illustrating the intersection between genes showed “nominal” significant differences in P/LP variants localization when CDS was split into 5 bins (5 bins), 2 parts (2 bins), and based on protein domains (domain boundaries).

Next, we hypothesized that the detection of differences in the localization of causal variants for distinct diseases associated with a common gene would be facilitated by considering the protein domain structure. Assuming that protein domains have different functions, one could expect that variants associated with different disorders could be in different domains of the same protein. Therefore, we tried to compare the number of P/LP variants associated with each disease that are in different domains of the same GMD (Figure 4a, see Section 2 for a more detailed description of the procedure). We anticipated that the test would identify more GMDs with significant differences in disease-causing variants abundance across domains. We were able to retrieve protein domain coordinates for 362 genes linked to exactly two rare diseases, and 2 or more domains were annotated for 217 of these genes (Figure 4b). Contrary to our expectations, only *FLT4* reached FDR-adjusted statistical significance in the test, and only 6 genes reached nominal significance (*ABCA12*, *CREBBP*, *HSD17B4*, *OCRL*, *SMARCA2*, *TEK*) (Supplementary Figure S4a). A slightly better result was achieved when removing the pleiotropic variants, with two genes exhibiting FDR-adjusted statistical significance (*CREBBP* and *FLT4*) and 6 genes showing nominal significance (*ABCA12*, *BTK*, *OCRL*, *PDE10A*, *SMARCA2*, and *TEK*) (Supplementary Figure S4b).

**Figure 5:**
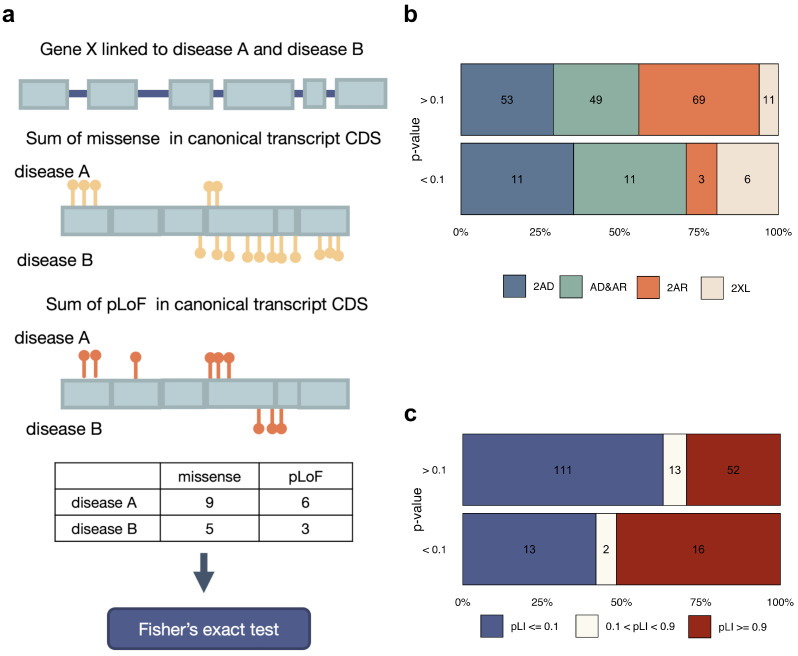
The influence of the variant type on the observed phenotype. (**a**) A schematic illustration of the workflow of analysis of variant type proportion within the coding sequence of the genes with 2 associated diseases. (**b**,**c**) The proportion of genes in different inheritance pattern categories (2AD, AD&AR, 2AR, 2XL) (**b**) or different LoF-tolerance groups (**c**) for genes with or without weak nominally significant (*p* < 0.1) differences in variant type proportions.

The last factor that may determine the development of a specific disease associated with GMDs is the type of causal variant. To test the relevance of this factor for phenotype determination, we compared the proportion of missense and pLoF variants between two diseases linked to the same GMD (Figure 5a, see Section 2 for a more detailed description of the procedure). As can be seen from the quantile-quantile plot in Supplementary Figure S2d, more substantial deviations from the expectation were observed in the variant type-based test compared to the variant localization-based case. In total, 13 genes reached FDR-adjusted statistical significance (*CAPN3*, *CDKN1C*, *COL4A3*, *CREBBP*, *FLT4*, *KCNQ2*, *KMT2D*, *NALCN*, *NOTCH2*, *SLC12A6*, *SLC26A4*, *SMARCA4*, *SMC1A*). The predominant type of causal variants for each disease linked to GMDs could be observed in Table 2. Insignificant association of the test result with the constraint metrics (*p*-value in chi-squared test = 0.0522) have been observed (Figure 5c). At the same time, differences in the type of causal variants also show a significant association with the mode of inheritance of associated diseases (*p*-value in chi-squared test = 0.0043) (Figure 5b), especially for genes linked to AD diseases, which is consistent with previous studies [23].

## 4. Discussion

Identifying the causal variant among hundreds of thousands detected by NGS methods remains a significant challenge in medical genetics. The issue is complicated by the phenotypic heterogeneity, which hinders the ascertainment of the link between pathogenic variant(s) and the observed phenotype. In our work, we made an effort to dissect the molecular mechanisms of this phenomenon by investigating the properties of genetic variation in GMDs.

Our analysis revealed that around 20% of Mendelian disease genes are associated with two or more diseases according to the OMIM/HPO data. These GMDs are more conserved compared to GSDs and are significantly enriched for genes involved in muscle contraction and dilated and hypertrophic cardiomyopathy pathways (Figure 1e,f). These findings corroborate previous studies of inherited cardiac disorders [6,8,24] and movements disorders [25].

To elucidate the origin of the phenomenon, we analyzed genetic factors that could influence the phenotypic outcome. Specifically, we examined the type of causal variant and its location within a coding sequence. These two factors are arguably the most clearly defined properties of a genetic variant; however, it appears likely that other variant features may play a role in phenotypic heterogeneity. For example, the exact conformational changes imposed by a variant may affect the function of the protein and, hence, be important for phenotypic heterogeneity [26]. However, estimation of the degree of influence of a variant on conformation requires a systematic study involving in silico protein folding tools.

The evaluation of the intragenic distribution of disease-specific variants in GMDs confirmed that variant localization is an important factor for phenotype determination in genes linked to two AD diseases. This observation is consistent with previous findings of pathogenic missense variants clustering, which was predominantly observed in single-disease genes linked to AD rather than AR disease [23]. The relationship between intragenic variant localization and phenotype is important for accurate variant annotation and prioritization. For example, some studies have described scores for improving the prediction of variant pathogenicity based on analysis of pathogenic variants clustering in regions encoding functional protein domains [27].

Importantly, our observations are in good concordance with several gene-level studies. One example is the *SRCAP* gene linked to Floating-Harbor syndrome (FHS, OMIM 136140), in which causal variants have been identified only in 33rd and 34th exons [28]. At the same time, pathogenic variants outside of this hotspot region have been described in association with developmental delay, hypotonia, musculoskeletal defects, and behavioral abnormalities (DEHMBA, OMIM 619595) [29]. Another notable example is the *CDKN1C* gene, which, among several others, showed FDR-adjusted significant differences in variant localization for the two corresponding diseases—the IMAGE syndrome (OMIM:614732) and the Beckwith–Wiedemann syndrome (OMIM:130650). IMAGE syndrome is caused by a mutation in a specific replication accessory protein binding domain, as reported by Arboleda et al., 2012 [30]. On the other hand, causal variants of Beckwith–Wiedemann syndrome are scattered across the coding sequence.

It is also important to note that we identified more genes with significant differences in variant localization between diseases when splitting the CDS into equally sized parts rather than actual protein domains. As protein domains usually have distinct functions, variants located in different domains should confer different molecular changes resulting in distinct phenotypic outcomes [31]. The lower number of significant genes in the domain boundary-based test of variant localization indicates that either (i) currently available data are insufficient to identify domain-specific localization of variants linked to different diseases or (ii) structural elements other than single domains contribute to phenotypic heterogeneity in GMDs. Still, our analysis identified domain-specific variant localization of disease-specific variants in some GMDs. For example, *FLT4* gene solely shows significant differences in P/LP variants distribution across domains. Although defects in the lymphatic system (OMIM:153100) are primarily caused by variants at the protein kinase domain, the variants leading to congenital heart defects are in 6 different domains, including Ig-like and kinase domains.

Our analysis indicates that variation in the proportions of missense and pLoF causal variants between two diseases linked to a single gene seems to be of relatively greater importance compared to variant localization (Supplementary Figure S2). Indeed, the variant type may modulate the phenotype by affecting the quantity or quantity of the gene product. Although missense variants are likely to result in quantitative changes in gene product level or its activity, pLoF variants are more likely to lead to complete loss of gene product function [25]. However, the exact relationship between these properties of genetic variants and specific traits in the phenotype remains to be established.

In summary, our analysis allowed us to dissect the determinants of heterogeneous gene–disease relationships for 49 out of 670 GMDs that are linked to exactly 2 conditions. We detect a significant impact of variant type on phenotype determination for 30 genes (Supplementary Table S2) and of variant localization—for 38 genes (Figure 4c, Supplementary Table S2). Notably, 19 GMDs with 2 associated disorders show differences both in variant localization and variant type proportions for the two associated diseases (Supplementary Table S2). We believe that the aforementioned results obtained in our analysis might be used to improve the quality of variant annotation and prediction of the variant’s pathogenicity.

It is important to note, however, that the overall power of our analysis of disease-specific variant localization was not sufficient to detect a substantial number of significant genes. It might be partially explained by limitations that arise when conducting analysis solely using public data. Our study was based only on genes and P/LP variants with reported associated rare diseases in ClinVar. However, a substantial fraction of P/LP variants are annotated as causal for all diseases associated with a specific gene (Figure 2), and the lack of correlation of such variants’ proportion with inheritance patterns or phenotypic similarities raises doubts about the validity of the variant–disease association. Moreover, even if data quality is not considered, the study remains biased toward existing knowledge about gene–disease and variant–disease associations, and some relevant information might be missed by such an approach. Hence, further studies based on larger curated datasets are necessary to comprehensively characterize the factors contributing to phenotypic heterogeneity in GMDs. It is also important to note that the determinants of phenotypic heterogeneity may be different in complex traits, in which genetic interactions and the environment play a much greater role. Thus, further integration of results obtained in monogenic and complex traits is required to fully understand the complex nature of genotype-to-phenotype relationships.

## Figures and Tables

**Table 1:** Genes that show significant differences (FDR-adjusted *p*-value < 0.05) in analysis of causal variant localization for 2 associated monogenic diseases.

Gene	Associated Disease	OMIM Identifier	CDS Quintiles with Causal Variants Prevalence ^†^
*CDKN1C*	Beckwith–Wiedemann syndrome	OMIM:130650	II, IV, V
	IMAGE syndrome	OMIM:614732	I
*CREBBP*	Rubinstein–Taybi syndrome 1	OMIM:180849	II, III, V
	Menke–Hennekam syndrome 1	OMIM:618332	II
*DEAF1* ^‡^	Neurodevelopmental disorder with hypotonia, impaired expressive language, and with or without seizures	OMIM:617171	II, IV
	Vulto–van Silfout–de Vries syndrome	OMIM:615828	III
*FLT4* ^‡^	Lymphatic malformation 1	OMIM:153100	I, II
	Congenital heart defects, multiple types, 7	OMIM:618780	I, IV
*FN1* ^‡^	Glomerulopathy with fibronectin deposits 2	OMIM:601894	II
	Spondylometaphyseal dysplasia, corner fracture type	OMIM:184255	V
*F8* ^‡^	Hemophilia A	OMIM:306700	I, V
	Thrombophilia 13, X-linked, due to factor VIII defect	OMIM:301071	I
*KMT2D* ^‡^	Branchial arch abnormalities, choanal atresia, athelia, hearing loss, and hypothyroidism syndrome	OMIM:620186	II
	Kabuki syndrome 1	OMIM:147920	I, II, IV
*MAF* ^‡^	Ayme-Gripp syndrome	OMIM:601088	II, V
	Cataract 21, multiple types	OMIM:610202	II
*NFIX* ^‡^	Marshall-Smith syndrome	OMIM:602535	I, IV
	Malan syndrome	OMIM:614753	I, II
*OCRL*	Dent disease 2	OMIM:300555	I, III
	Lowe syndrome	OMIM:309000	II, III
*SETBP1* ^‡^	Intellectual developmental disorder, autosomal dominant 29	OMIM:616078	II
	Schinzel-Giedion midface retraction syndrome	OMIM:269150	III
*SPTB*	Spherocytosis type 2	OMIM:616649	II, IV, V
	Elliptocytosis-3	OMIM:617948	I
*SRCAP* ^‡^	Developmental delay, hypotonia, musculoskeletal defects, and behavioral abnormalities	OMIM:619595	-
	Floating-Harbor syndrome	OMIM:136140	IV

^†^ Coding region in which causal variants are more prevalent for a particular disease compared to a uniform expectation. ^‡^ Genes that show significant differences in localization of causal variants only after removal of pleiotropic variants from the dataset.

**Table 2:** Genes that show significant differences (FDR-adjusted *p*-value < 0.05) in analysis of causal variant type proportions for 2 associated monogenic diseases.

Gene	Associated Disease	OMIM Identifier	Causal Variant Count	
			Missense	pLoF
*CAPN3*	Muscular dystrophy, limb-girdle, autosomal recessive 1	OMIM:253600	67	109
	Muscular dystrophy, limb-girdle, autosomal dominant 4	OMIM:618129	15	3
*CDKN1C*	Beckwith–Wiedemann syndrome	OMIM:130650	5	55
	IMAGE syndrome	OMIM:614732	7	1
*COL4A3*	Alport syndrome 3, autosomal dominant	OMIM:104200	68	23
	Alport syndrome 2, autosomal recessive	OMIM:203780	51	65
*CREBBP*	Rubinstein–Taybi syndrome 1	OMIM:180849	8	76
	Menke–Hennekam syndrome 1	OMIM:618332	5	1
*FLT4*	Lymphatic malformation 1	OMIM:153100	13	0
	Congenital heart defects, multiple types, 7	OMIM:618780	0	7
*KCNQ2*	Seizures, benign neonatal, 1	OMIM:121200	69	25
	Developmental and epileptic encephalopathy 7	OMIM:613720	115	13
*KMT2D*	Kabuki syndrome 1	OMIM:147920	42	221
	Branchial arch abnormalities, choanal atresia, athelia, hearing loss, and hypothyroidism syndrome	OMIM:620186	6	0
*NALCN*	Hypotonia, infantile, with psychomotor retardation and characteristic facies 1	OMIM:615419	5	11
	Congenital contractures of the limbs and face, hypotonia, and developmental delay	OMIM:616266	31	2
*NOTCH2*	Hajdu-Cheney syndrome	OMIM:102500	0	15
	Alagille syndrome-2	OMIM:610205	5	1
*SLC12A6*	Agenesis of the corpus callosum with peripheral neuropathy	OMIM:218000	0	42
	Charcot-Marie-Tooth disease, axonal, type 2	OMIM:620068	5	0
*SLC26A4*	Pendred syndrome	OMIM:274600	71	49
	Deafness, autosomal recessive 4, with enlarged vestibular aqueduct	OMIM:600791	96	21
*SMARCA4*	Rhabdoid tumor predisposition syndrome-2	OMIM:613325	2	38
	Coffin-Siris syndrome-4	OMIM:614609	8	0
*SMC1A*	Cornelia de Lange syndrome 2	OMIM:300590	49	21
	Developmental and epileptic encephalopathy 85, with or without midline brain defects	OMIM:301044	2	10

## Data Availability

The data and code pertinent to the analysis presented in the current work are available at https://github.com/tanya-lazareva/phenohet.git (accessed on 8 October 2023).

## Supplementary Materials

The following supporting information can be downloaded at: https://www.mdpi.com/article/10.3390/genes14112100/s1, Figure S1: Phenotypic similarity between 2 diseases associated with one common gene; Figure S2: Results of statistical analysis of within-gene distribution and type of variants; Figure S3: Distribution of phenotypic similarity in genes with and without significant differences in variant localization between associated diseases; Figure S4: Results of statistical analysis of variant distribution within protein domain boundaries; Table S1: GMDs linked to exactly 2 conditions with more than 50% of shared causal variants.; Table S2: List of GMDs with significant and nominally significant differences in variant type proportions of variants for the two associated diseases; variant localization for the two associated diseases.

## Abbreviations

The following abbreviations are used in this manuscript:

HPO	Human Phenotype Ontology
NGS	next-generation sequencing
OMIM	Online Mendelian Inheritance in Man
P/LP	pathogenic/likely pathogenic variants
pLoF	predicted loss-of-function variant
WES	whole-exome sequencing
WGS	whole-genome sequencing
