# Supplementary material for: Statistical Dissection of the Genetic Determinants of Phenotypic Heterogeneity in Genes with Multiple Associated Rare Diseases
Source: Genes (Basel). 2023 Nov 18;14(11):2100. doi: 10.3390/genes14112100 (PMC10671084; doi:10.3390/genes14112100)
Supplement: Supplementary file 1 [file genes-14-02100-s001.zip › Supplementary.FigsS1-S4.pdf]

# Statistical Dissection of the Genetic Determinants of Phenotypic Heterogeneity in Genes with Multiple AssociatedRare Diseases

**Tatyana E. Lazareva 1, Yury A. Barbitoff 1,2,* , Yulia A. Nasykhova 1 , Nadezhda S. Pavlova 2, Polina M. Bogaychuk 2 and Andrey S. Glotov 1,***

[1] Department of Genomic Medicine, D.O. Ott Research Institute of Obstetrics, Gynaecology, and
[2] Reproductology, 199034 St. Petersburg, Russia; lazata1997@gmail.com (T.E.L.); yulnasa@gmail.com (Y.A.N.)
[3] Bioinformatics Institute, Kantemirovskaya St. 2A, 197342 St. Petersburg, Russia; pav.nad.ser@gmail.com (N.S.P.); pm.bogaichuk@gmail.com (P.M.B.)
* Correspondence: barbitoff@bk.ru (Y.A.B.); anglotov@mail.ru (A.S.G.)

## Supplementary information

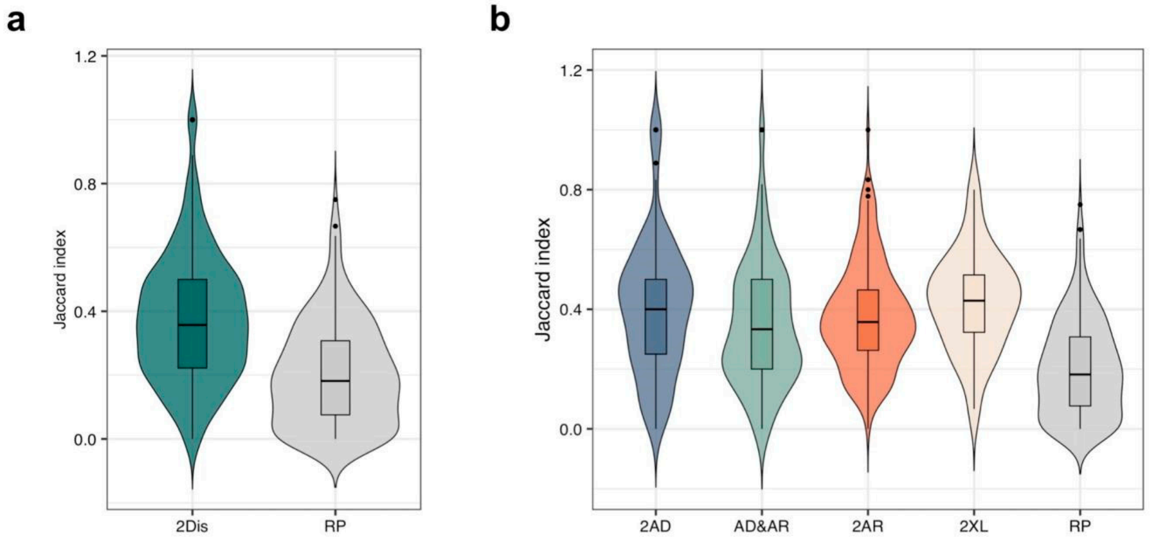


**Figure S1.** Phenotypic similarity between 2 diseases associated with one common gene. (a) Phenotypic similarity between 2 disorders linked to the same gene compared to random disease pairs (RP). (b) Phenotypic similarity between the two diseases linked to the same gene in each inheritance pattern group. Similarity between two randomly chosen diseases (RP) is shown as a baseline for comparison.

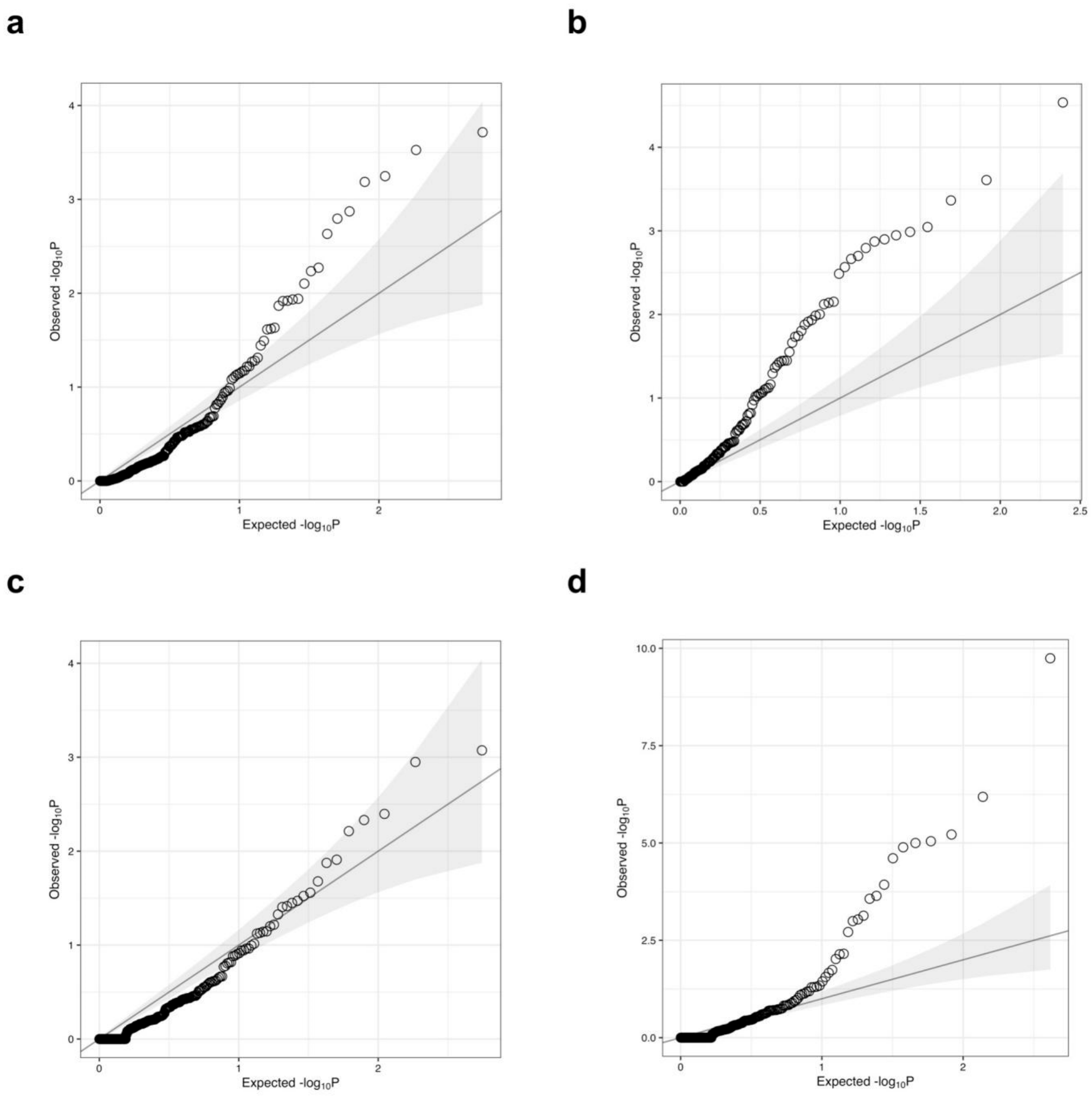


**Figure S2** Results of statistical analysis of within-gene distribution and type of variants. Quantile-quantile plot showing the results of the statistical comparison of localization of (a) all P/LP variants; (b) disease-specific P/LP variants associated with each of the two diseases in the 5-bin test; and (c) all P/LP variants in the 2-bin tests. (d) Quantile-quantile plot showing the results of the statistical comparison of the proportion of missense and pLoF variants associated with each of the two diseases for GMDs linked to exactly 2 conditions. Expected p-values are drawn from the uniform distribution, observed p-values correspond to the Fisher's exact test p-values.

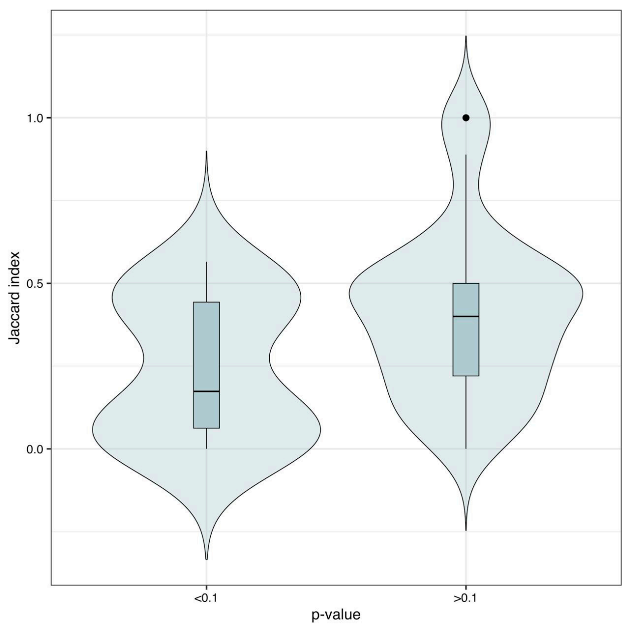


**Figure S3** Distribution of phenotypic similarity in genes with and without significant differences in variant localization between associated diseases. Plots showing the distribution of the Jaccard index of upper-level HPO terms for genes with ("significant") or without ("not significant") weak nominally significant ($p < 0.1$) differences in distribution of variants for the two associated diseases within splitted on 5 bins CDS.

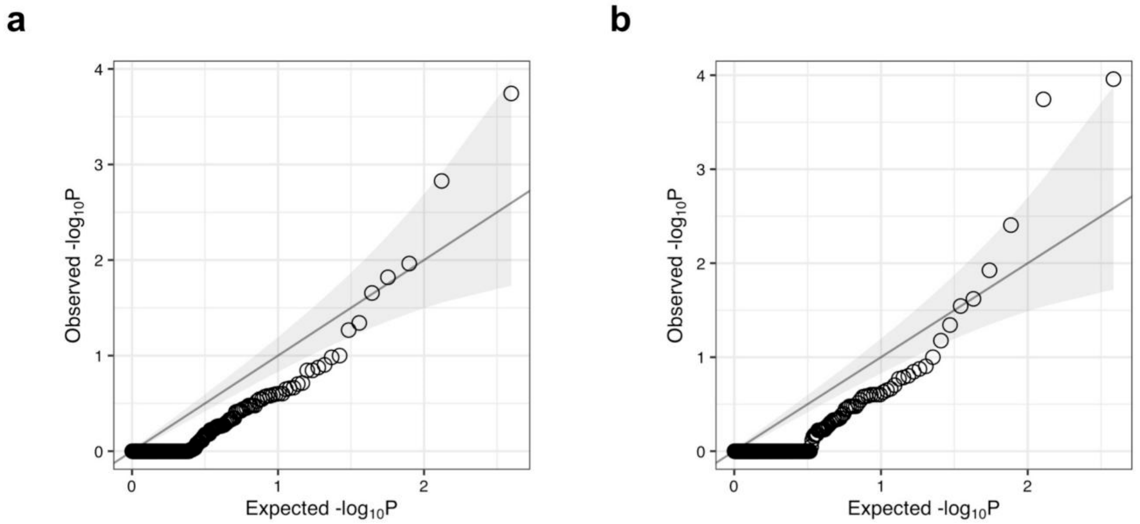


**Figure S4** Results of statistical analysis of variant distribution within protein domain boundaries. Quantile-quantile plot showing the results of the statistical comparison of localization of (a) all P/LP variants (b) disease-specific P/LP variants associated with each of the two diseases for split based on protein domain genomic boundaries.
